# Do young adults with cancer receive information about treatment‐related impact on sex life? Results from a population‐based study

**DOI:** 10.1002/cam4.5672

**Published:** 2023-02-07

**Authors:** Charlotta Bergström, Claudia Lampic, Ricky Roy, Christel Hedman, Johan Ahlgren, Olof Ståhl, Karin E. Smedby, Kristina Hellman, Roger Henriksson, Lars E. Eriksson, Lena Wettergren

**Affiliations:** ^1^ Department of Women's and Children's Health Karolinska Institutet Stockholm Sweden; ^2^ Department of Surgery and Urology Danderyd Hospital Stockholm Sweden; ^3^ Department of Public Health and Caring Sciences Uppsala University Uppsala Sweden; ^4^ Department of Psychology Umeå University Umeå Sweden; ^5^ Department of Urology Karolinska University Hospital Huddinge Sweden; ^6^ Department of Molecular Medicine and Surgery Karolinska Institutet Stockholm Sweden; ^7^ R&D Department Stockholms Sjukhem Foundation Stockholm Sweden; ^8^ Department of Clinical Sciences Lund Lund University Lund Sweden; ^9^ Department of Oncology Faculty of Medicine and Health Örebro University Örebro Sweden; ^10^ Regional Cancer Center, Mid‐Sweden Uppsala Sweden; ^11^ Department of Oncology Skåne University Hospital Lund Sweden; ^12^ Department of Medicine Solna Clinical Epidemiology Division Karolinska Institutet Stockholm Sweden; ^13^ Department of Hematology Karolinska University Hospital Stockholm Sweden; ^14^ Department of Gynecologic Cancer Theme Cancer Karolinska University Hospital Stockholm Sweden; ^15^ Department of Radiation Science and Oncology University Hospital Umeå Sweden; ^16^ Department of Neurobiology, Care Sciences and Society Karolinska Institutet Huddinge Sweden; ^17^ School of Health and Psychological Sciences City, University of London London UK; ^18^ Medical Unit Infectious Diseases Karolinska University Hospital Huddinge Sweden

**Keywords:** communication, health personnel, neoplasms, sexual dysfunction, young adult

## Abstract

**Background:**

Sexual dysfunction is common following a cancer diagnosis in young adulthood (18–39 years) and problems related to sex life are ranked among the core concerns in this age group. Yet, few studies have investigated to what extent adults younger than 40, receive information from healthcare providers about the potential impact of cancer and its treatment on their sex life.

**Methods:**

A population‐based cross‐sectional survey study was conducted with 1010 young adults 1.5 years after being diagnosed with cancer (response rate 67%). Patients with breast, cervical, ovarian and testicular cancer, lymphoma, and brain tumors were identified in national quality registries. Sociodemographic and clinical factors associated with receiving information were examined using multivariable binary logistic regression.

**Results:**

Men to a higher extent than women reported having received information about potential cancer‐related impact on their sex life (68% vs. 54%, *p* < 0.001). Receipt of information varied across diagnoses; in separate regression models, using lymphoma as reference, both women and men with brain tumors were less likely to receive information (women: OR 0.10, CI = 0.03–0.30; men: OR 0.37, CI = 0.16–0.85). More intensive treatment was associated with higher odds of receiving information in both women (OR 1.89; CI = 1.28–2.79) and men (OR 2.08; CI = 1.09–3.94). None of the sociodemographic factors were associated with receipt of information.

**Conclusions:**

To improve sexual health communication to young adults with cancer, we recommend diagnosis‐specific routines that clarify when in the disease trajectory to discuss these issues with patients and what to address in these conversations.

## Graphical abstract

Young women with cancer report having received information about potential treatment‐related impact on sex life to a lesser extent than men, and there is also a variation across cancer types. The majority of recipents recalled being informed that there was "some risk" of sex life being affected, see figure below. Routines clarifying when to discuss these issues are recommended to improve sexual health communication with young patients in cancer care.

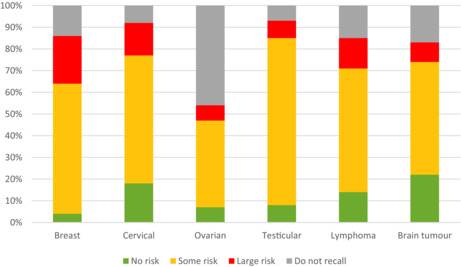

## 1 INTRODUCTION

Nearly 1 million young adults worldwide, commonly defined as those between ages of 18–39,^1^ are diagnosed with cancer every year.^1^ Cancer and its treatment can cause various sexual problems.^2^ Commonly reported issues include vaginal dryness, low interest in having sex, erectile dysfunction, and decreased satisfaction with sex life.^2^ Previous research indicates that approximately 50% of patients report sexual problems the first 2 years following a cancer diagnosis in young adulthood.^3^, ^4^

Sexuality is an integral part of life.^5^ Sexual problems are ranked among the core concerns in young adults with cancer, a period in life with intimate relationships and family building. Dating following cancer has been reported to be challenging. Young patients with limited experience of sex with a partner before getting cancer may be unsure about what experiences that are expected and considered normal.^6^, ^7^ In addition, worries about fertility‐related issues can affect sexuality negatively.^8^ Studies have pinpointed unmet needs of support and information in this area.^6^, ^7^, ^9^

National^10^ as well as international guidelines^11^ recommend that health care providers address sexual health and dysfunction with all patients diagnosed with cancer. For patients with high risk of treatment‐related impact on their sex life, adequate information may prepare them to handle negative side effects. For patients whose treatment pose little or no risk of impact on sex life, information about this is equally important as they otherwise may be unnecessarily worried. A systematic review of 29 studies from 10 countries found that among adult cancer populations (>40 years at diagnosis), on average 60% of men and 28% of women reported that the cancer treatment's potential impact on their sex life had been communicated.^12^ Receipt of information has shown to be positively associated with male gender and reproductive cancers, such as prostate and gynecological cancer.^12^, ^13^, ^14^

To what extent young adults diagnosed with cancer receive information from the healthcare regarding potential impact on their sex life is not fully understood. Most studies include patients of all ages, making it difficult to draw conclusions regarding the minority group diagnosed before the age of 40. According to the results of the few previous studies (*n* = 4) that specifically focused on young adults, between a third and half of them (33%–52%) reported that they had been informed or discussed potential impact on sex life with a healthcare provider.^15^, ^16^, ^17^, ^18^ However, these studies are heterogeneous with regard to patient age (15–49 years), time since diagnosis (1–7 years post‐diagnosis), and how the question was expressed, which limits the possibility to draw firm conclusions. Furthermore, the sampling strategies with only one population‐based study^15^ and predominantly low response rates (42%–52%)^15^, ^16^, ^18^ contribute to a somewhat imprecise picture of the situation. The aim of the present study was therefore to investigate to what extent young adults recall receiving information about possible impact of cancer and its treatment on sex life approximately 18 months after being diagnosed with cancer, using a population‐based sample. Further, we aimed to identify potential sociodemographic and clinical factors associated with having received such information.

## 2 MATERIALS AND METHODS

### 2.1 Study design

The present study is part of the population‐based Fertility and Sexuality following cancer (Fex‐Can) Cohort study.^19^ The study monitors sexual function and fertility‐related distress in young women and men up to 5 years after being diagnosed with cancer. The current report presents cross‐sectional data from the baseline assessment (1.5 years after diagnosis), conducted 2017–2019. Details on recruitment and methods have been described in detail previously^19^ and are described in short below in accordance with STROBE guidelines.^20^ Ethical approval was obtained from the Regional Ethical Review Board in Stockholm (record no: 2013/1746‐31/4; 2014/2244‐32; 2017/916‐32; 2017/1416‐32).

### 2.2 Study population

Inclusion criteria were as follows: diagnosed at age 18–39 years with breast cancer (women only), testicular, ovarian or cervical cancer, lymphoma, or brain tumor, during the period January 2016 through August 2017. The diagnoses were selected since they are relatively common in this age interval and known to potentially affect sexual function and/or fertility. All patients were identified through national quality registers, and those meeting the criteria were approached approximately 1.5 years after diagnosis and asked to complete a comprehensive survey. Informed consent was obtained from all participants prior to their inclusion.

### 2.3 Outcomes

Receipt of information was evaluated through the study‐specific question, “Have you received information from the healthcare on whether your cancer or cancer treatment could affect your sex life?” with three response alternatives (No/Yes/Do not know or Do not recall). Participants responding affirmatively answered two additional questions: “If yes, from whom?” (Physician/Nurse/Other health care provider/Brochure) and “If yes, what were you told about the risk of your sex life being affected?” (None/Some risk/High risk/Do not recall). Several response alternatives could be selected.

### 2.4 Sociodemographic characteristics and clinical data

Sociodemographic characteristics were collected in the survey and clinical data were retrieved from the national quality registries. Based on diagnosis, stage and type of treatment, individuals' treatment intensity was classified (least/moderate/very/most) according to the Intensity Treatment Rating scale ‐Young Adult (ITR‐YA).^21^

### 2.5 Statistical analysis

Statistical analyses were conducted with SPSS Statistics for Windows, version 28 (IBM Corp). Student's *t*‐test and chi‐square test were used for group comparisons (responders/non‐responders and men/women).

Sociodemographic variables and treatment intensity were categorized: Birth country (Sweden/other country); Education (university/non‐university); Sexual orientation (heterosexual/non‐heterosexual); Partner status (partnered/non‐partnered); Have children (yes/no); Age at diagnosis (18–29/30–35/ 36–39); and Treatment intensity (least, moderate/, very, most). Factors associated with receipt of information were identified using multivariable binary logistic regression models expressed as odd ratios (OR) using 95% confidence intervals (CI). The factors were selected beforehand according to previous findings in the literature: age,^22^, ^23^ diagnosis,^13^, ^17^, ^24^ country of birth,^23^ education,^25^ sexual orientation,^26^, ^27^ treatment intensity,^22^, ^25^ and current partnership.^13^ Due to a strong correlation between the variables gender and cancer diagnosis, the analyses were stratified by gender. For all tests, *p* < 0.05 was considered statistically significant.

## 3 RESULTS

### 3.1 Participants

Of the 1499 approached individuals, 1010 completed the survey, 694 women and 316 men (response rate 67%). Comparison of responders and non‐responders showed statistically significant differences, as previously reported.^28^ In short, women participated to a higher extent than men (*p* < 0.0001), and participation rate in women differed by cancer type, ovarian cancer 56%, brain tumors 62%, lymphoma 72%, cervical cancer 73%, and breast cancer 75% (*p* = 0.006). Male responders were older than non‐responders (*p* = 0.001). Among the participants, the mean age at diagnosis was 32 (range 18–39), and the most common cancer diagnoses were breast cancer (35%) and testicular cancer (20%). Additional sociodemographic and clinical data of the participants are presented in Table 1.

**TABLE 1:** Sociodemographic and clinical characteristics of study participants.

Characteristics	Total (*N* = 1010), *n* (%)	Women (*n* = 694), *n* (%)	Men (*n* = 316), *n* (%)
Age at diagnosis (years)			
18–29	288 (28)	157 (23)	131 (41)
30–35	362 (36)	250 (36)	112 (35)
36–39	360 (36)	287 (41)	73 (23)
Diagnosis			
Breast cancer	349 (35)	349 (50)	—
Cervical cancer	190 (19)	190 (27)	—
Ovarian cancer	32 (3)	32 (5)	—
Testicular cancer	200 (20)	—	200 (63)
Lymphoma	116 (11)	57 (8)	59 (19)
Brain tumor	123 (12)	66 (10)	57 (18)
Birth country^a^			
Sweden	851 (84)	579 (83)	272 (86)
Other country	157 (16)	114 (16)	43 (14)
Highest education^a^			
University	559 (55)	417 (60)	142 (45)
Other education level	449 (45)	275 (40)	174 (55)
Main occupation^a^			
Working or studying	799 (79)	530 (77)	269 (85)
Unemployed, sick leave, other^b^	205 (21)	162 (23)	47 (15)
Sexual orientation^a^			
Heterosexual	930 (94)	633 (93)	297 (96)
Non‐heterosexual	59 (6)	45 (7)	14 (4)
Relationship status^a^			
Partnered	830 (82)	585 (85)	245 (78)
Not Partnered	178 (18)	107 (15)	71 (22)
Have children			
Yes	621 (61)	473 (68)	148 (47)
Intensity of treatment^a^ ^,^ ^c^			
Least/moderate	500 (51)	314 (47)	186 (60)
Very/most	485 (49)	359 (53)	126 (40)

Abbreviation: SD, Standard deviation.

^a^ Does not sum up to total due to missing data.

^b^ Parental leave or retired.

^c^ According to the Intensity of Treatment Rating Young Adult (ITR‐YA).

### 3.2 Received information on potential impact on sex life

Receipt of information was reported by 54% of the women and 68% of the men (*χ* ^2^ = 17.09, *p* < 0.001). The proportions varied across cancer diagnoses, men with testicular cancer reported the highest receipt of information (78%) followed by women with breast or cervical cancer (61%), see Table 2. Among patients diagnosed with lymphoma and brain tumors, women reported significantly lower receipt of information than men (lymphomas 46% vs. 64%, *p* < 0.05; brain tumors 6% vs. 33%, *p* < 0.001).

**TABLE 2:** Receipt of information as reported by young adults with cancer.

Diagnosis	Receipt of information, *n* (%)	No information,^a^ *n* (%)	df	*𝝌* ^2^	*p*‐value
Women (*n* = 690)					
Breast cancer	211 (61)	136 (39)	4	73.01	<0.001
Cervical cancer	114 (61)	74 (39)			
Ovarian cancer	15 (47)	17 (53)			
Lymphoma	26 (46)	31 (54)			
Brain tumor	4 (6)	62 (94)			
Men (*n* = 314)					
Testicular cancer	155 (78)	43 (22)	2	41.09	<0.001
Lymphoma	38 (64)	21 (36)			
Brain tumor	19 (33)	38 (67)			

*Note*: Missing responses for four women and two men.

Abbreviation: df, degrees of freedom.

^a^ Including participants who answered no/do not recall any receipt of information.

**FIGURE 1:**
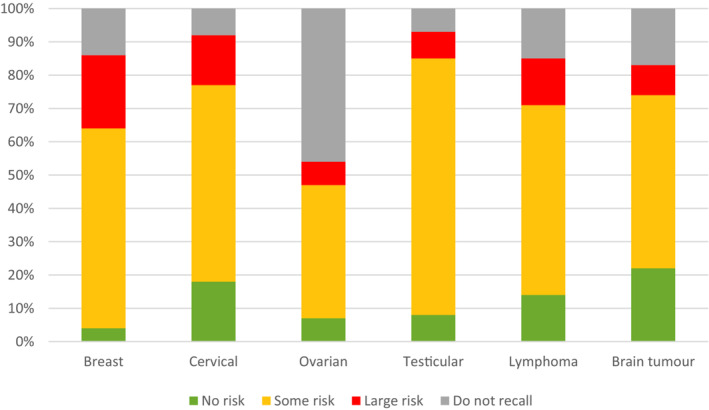
Risk of impact on one's sex life as reported by young adults with cancer. Those who reported having received information from the healthcare answered the following question: “If yes, what were you told about the risk of your sex life being affected?” (None/Some risk/High risk/Do not recall).

Among those who recalled having received information about impact on sex life (*n* = 582), the majority had received information from two or more of the following sources: physician (66%), brochure (53%), nurse (47%), other healthcare provider (9%). The majority of recipients recalled being informed that there was “some risk” of sex life being affected with the pattern of risk information varying across diagnoses. Notably, among the women with ovarian cancer who stated having received sex‐related information, half did not report any specific risk information received (Figure 1).

### 3.3 Factors associated with receiving information on possible impact on sex life

Receipt of information was associated with type of cancer and treatment intensity as shown in separate logistic regression models for women and men (Table 3). When lymphoma was used as reference group, women with cervical cancer were more likely to report having received information (OR = 2.42, 95% CI: 1.26–4.67), while women with brain tumors were less likely (OR = 0.10, 95% CI: 0.03–0.30). Among men, when lymphoma was used as reference group, patients with testicular cancer to a larger extent reported that they had received information (OR = 3.09, 95% CI: 1.43–6.69), and men with brain tumors to a lesser extent (OR = 0.37, 95% CI: 0.16–0.85). More intensive treatment was associated with higher odds of receipt of information in both women and men (OR = 1.89, 95% CI: 1.28–2.79; OR = 2.08, 95% CI: 1.09–3.94, respectively). None of the analyzed sociodemographic factors were associated with receipt of information.

**TABLE 3:** Factors associated with receipt of information about disease‐related impact on sex life in young women and men with cancer.

Factors	Women		Men	
	OR	95% CI	OR	95% CI
Age at diagnosis				
18–29	1		1	
30–35	0.98	0.60–1.57	1.07	0.57–1.99
36–39	0.88	0.54–1.43	0.71	0.37–1.39
Birth country				
Other	1		1	
Sweden	1.03	0.65–1.64	0.68	0.30–1.52
Highest education				
Other	1		1	
University	1.38	0.98–1.94	1.04	0.61–1.77
Sexual orientation				
Heterosexual	1		1	
Non‐heterosexual	1.24	0.64–2.40	1.65	0.43–6.30
Relationship status				
Non partnered	1		1	
Partnered	1.31	0.83–2.09	1.77	0.95–3.30
Diagnosis				
Lymphoma	1		1	
Breast cancer	1.72	0.93–3.20	—	—
Cervical cancer	**2.42**	1.26–4.67	—	—
Ovarian cancer	1.72	0.68–4.34	—	—
Brain tumor	**0.10**	0.03–0.30	**0.37**	0.16–0.85
Testicular cancer	—	—	**3.09**	1.43–6.69
Treatment intensity^a^				
Least/moderate	1		1	
Very/most	**1.89**	1.28–2.79	**2.08**	1.09–3.94

*Note*: Statistically significant (*p* < 0.05) factors in the multivariable models indicated in bold. Difference in sample size due to missing data from registered treatment intensity, ITR (women *n* = 21, men *n* = 4) and item responses (women *n* = 21 and men *n* = 8).

Abbreviations: CI, confidence interval; OR, odd ratio.

^a^ According to the Intensity Treatment Rating Young Adult (ITR‐YA).

## 4 DISCUSSION

In this nationwide population‐based study of young patients with cancer, approximately half of the women and two thirds of the men recalled having received some information about potential impact of the cancer and its treatment on their sex life. Women reported having received information to a lower extent than men did, and patients with brain tumors, regardless of gender, reported that they had been informed to a lesser extent than those with other cancers. Treatment intensity was also associated with receipt of information, patients who underwent less intensive treatment were at higher risk of not receiving information.

The prevalence of men (68%) and women (54%) who recalled having received information about cancer‐related impact on sex life, is higher than in previous studies of young adults with cancer.^15^, ^16^, ^17^, ^18^ Two of these studies focused on women, and reported receipt of information in 52% of women with breast cancer^16^ (vs. our results on breast cancer 61%) and 40% of women with breast, gynecological, or hematological cancer.^17^ Additionally, in a large population‐based study of young women and men (15–29 years), 33% had discussed how their cancer may affect their love‐life or sex life, with a health care professional.^15^ Thus, our prevalence rates exceed earlier findings by 10% or more and suggest increased attention to sexual health issues in current Swedish cancer care. However, the phrasing of the questions about sex‐related communication and the follow‐up time after diagnosis varied across studies, making it difficult to draw firm conclusions regarding reasons for differences in study results.

The present results indicate that there is room for improvement in how sexual issues are communicated in healthcare, particularly to female patients, and to both men and women with brain tumors. Our findings of gender differences are in line with previous results among adults diagnosed after the age of 40.^12^, ^13^, ^22^, ^24^ while studies focusing on young adults have not reported comparative analysis between the genders. Also, no studies have presented results on sex information separately for patients with brain tumors.

Self‐reported receipt of information may be related to both internal and external factors. Characteristics of the healthcare providers such as their knowledge and perceptions are included in internal factors. Physicians and nurses who do not feel comfortable talking about sex may hesitate to raise the subject, and assumptions about the patient's wish to discuss the subject could affect if the issue is addressed.^29^, ^30^ Uncertainty on treatment options in some diagnostic groups may contribute, for example, women diagnosed with breast cancer are usually treated with anti‐estrogens and it may be more complicated to alleviate their symptoms of sexual dysfunction. Furthermore, healthcare providers may find it easier to inform when effective treatment options are available, for example when men worried about erectile dysfunction can be offered the well‐established PDE5‐inhibitors. Moreover, due to poor prognosis the subject may sometimes deliberately be left out. Regarding the low level of self‐reported information in patients with brain tumors, lack of knowledge about how a brain tumor and its treatments' may impact on sex life is likely to be an explanation. A recent published study on patients with brain tumors (age 19–84) found that sexual dysfunction was common.^31^ Only few studies with small samples have investigated this  among young adults with brain tumors, yet some indicate that sex problems are prevalent in patients with low‐grade glioma.^32^, ^33^ The women with brain tumors in the present cohort however did not report more sexual problems than women of a comparison group.^34^ The awareness of the risk of impaired cognitive function,^32^ may have led clinicians to leave out some information. Cognitive capacity was not evaluated in the present study but participants were at least able to complete our comprehensive survey at a time point 18 months after diagnosis.

External factors relate to the organization of care and include existing guidelines, access to educational material, and clinicians' working conditions, that is, enough time to discuss the side effects of treatment. National recommendations for how and when to address sex life vary between guidelines and is less developed for brain tumors than for gynecologic, and testicular cancer. For patients with testicular cancer, retrograde ejaculation can occur after lymph node dissection and measurement of testosterone levels is standard,^35^ and a discussion about sexual function may follow more easily.

The explanation for the discrepancy between men and women calls for attention, especially as previous studies of young adults show that sexual dysfunction following cancer is more prevalent among women.^25^, ^36^ Therefore, future studies should investigate how these discussions can be facilitated, especially among women. Our study revealed that patients with more intense treatment were more likely to report having received information about impact on sex life, indicating that those with higher risk of sexual impact from treatment also received more information. A considerable proportion of patients with ovarian cancer reported low level of self‐reported information; these women typically receive less intensive treatment with fertility sparing procedures, and the majority of young patients (<30 years old) have good prognosis. Future studies are recommended to investigate patients' satisfaction with the information they received and how communication can be improved.

International^11^ and national guidelines^10^ recommend that discussions about sexual health and dysfunction should be held by a member of the team treating the patient. Still, the present and previous results indicate that substantial groups of young adult patients do not recall sexual issues being addressed, emphasizing the need to facilitate sexual health discussions. A recent study indicated that there is a knowledge gap among oncologists about cancer drugs adverse effects on sexual function.^37^ Further, previous studies have shown that nurses and physicians inquire more knowledge and training in addressing the subject.^30^, ^37^ Interventions to improve communication skills about sex have shown promising results in a recently published review.^38^ To facilitate discussions about possible cancer‐related impact on sex life we recommend clinical routines, including checklists, to specify when in the disease trajectory these issues should be brought up with patients, and what to include in these conversations. Such routines need to be developed for each diagnosis to fit into the current care and type of treatment plan, and reflected in guidelines. Moreover, easy access to brochures about sexual health and cancer in waiting rooms could facilitate patient‐provider discussions as well as inform the patients that these problems are common and can be discussed with health care providers.

The major strengths of this study include the design with a large population‐based sample and inclusion of several diagnoses, drawn from the national cancer quality registers, lowering the risk of selection bias. Despite the sensitive topic, an overall acceptable response rate (67%) was achieved. However, men participated to a lesser extent than women, and women with ovarian cancer and brain tumors to a lesser extent than women with other cancer types. This limits the possibility to draw firm conclusions about these groups' situation, and emphasizes the need for further studies in men and certain groups of women. Further, participants with background other than Swedish were underrepresented (15% reported being born outside Sweden, compared to the corresponding foreign born proportion in Sweden of corresponding age, 25%),^39^ which might be explained by language barriers as the survey was only available in Swedish. Additionally, it is well known that patients' remembrance of medical information may be negatively influenced by stress and anxiety^40^ and recall bias may thus have resulted in underreporting of received information. However, as we recently found similarly high prevalence rates (≈80%) of received fertility‐related information among women and men in the present sample,^28^ we believe recall bias to have a limited effect on the present results. Our posed question assessed if information had been received, hence our results do not provide any information regarding satisfaction with information and whether a discussion took place or not. We therefore recommend future studies to explore cancer patients' satisfaction with such discussions.

In conclusion, this population‐based study of 1010 patients diagnosed with cancer in young adulthood found that nearly half of the women and one third of the men did not recall receiving information about potential impact on sex life. Furthermore, the proportion of patients who reported receipt of information varied across the diagnoses. To improve sexual health communication to young adults with cancer, we recommend diagnosis‐specific routines that clarify when these issues should be discussed with patients and what to address in these conversations.

## AUTHOR CONTRIBUTIONS

**Charlotta Bergström:** Formal analysis (equal); methodology (equal); visualization (lead); writing – original draft (equal); writing – review and editing (lead). **Claudia Lampic:** Conceptualization (equal); data curation (lead); funding acquisition (equal); investigation (equal); methodology (equal); project administration (equal); resources (equal); validation (equal); visualization (supporting); writing – review and editing (supporting). **Ricky Roy:** Formal analysis (equal); methodology (equal); visualization (lead); writing – original draft (equal); writing – review and editing (supporting). **Christel Hedman:** Methodology (equal); writing – review and editing (supporting). **Johan Ahlgren:** Methodology (equal); writing – review and editing (supporting). **Olof Ståhl:** Methodology (equal); writing – review and editing (supporting). **Karin Smedby:** Methodology (equal); writing – review and editing (supporting). **Kristina Hellman:** Methodology (equal); writing – review and editing (supporting). **Roger Henriksson:** Methodology (equal); writing – review and editing (supporting). **Lars E. Eriksson:** Conceptualization (supporting); data curation (supporting); investigation (equal); methodology (equal); supervision (supporting); visualization (supporting); writing – review and editing (supporting). **Lena Wettergren:** Conceptualization (equal); data curation (lead); funding acquisition (equal); investigation (equal); methodology (equal); project administration (equal); resources (equal); supervision (lead); validation (equal); visualization (supporting); writing – review and editing (lead).

## FUNDING INFORMATION

The Swedish Cancer Society (CAN 2013/886, CAN 2016/615, 190196Pj); the Swedish Childhood Cancer Foundation (TJ2014‐0050); the Cancer Research Funds of Radiumhemmet (161272), the Swedish Research Council for Health, Working Life and Welfare (2014‐4689, 2019‐00839); the Swedish Research Council (2017‐01530), the Vårdal Foundation (2014‐0098), and Karolinska Institutet's faculty funding for doctoral education (KID 2‐3591/2014).

## CONFLICT OF INTEREST STATEMENT

The authors have no conflicts of interest to disclose.
